# Identification of hub genes associated with esophageal cancer progression using bioinformatics analysis

**DOI:** 10.3892/ol.2020.12077

**Published:** 2020-09-09

**Authors:** Jiangfen Li, Yufang Xie, Xueli Wang, Chenhao Jiang, Xin Yuan, Anzhi Zhang, Lan Yang, Chunxia Liu, Hong Zou, Feng Li, Jianming Hu

**Affiliations:** 1 Department of Pathology and Key Laboratory for Xinjiang Endemic and Ethnic Diseases (Ministry of Education), The First Affiliated Hospital, Shihezi University School of Medicine, Shihezi, Xinjiang 832000, P.R. China; 2 Department of Pathology, Beijing Chaoyang Hospital, Capital Medical University, Beijing 100000, P.R. China

**Keywords:** esophageal cancer, hub genes, expression profiling data, ubiquitin conjugating enzyme E2 C, cyclin dependent kinase inhibitor 3, CDC28 protein kinase regulatory subunit 2, kinesin family member 20A, RAD51 associated protein 1

## Abstract

The underlying causes of esophageal cancer (EC) are unknown. To explore the molecular mechanisms that lead to EC, gene expression profiles of large cohorts of patients with EC were obtained from The Cancer Genome Atlas and the Gene Expression Omnibus (GEO) databases (GSE5364, GSE20347 and GSE23400). The present study identified 83 upregulated and 22 downregulated genes between EC and normal tissue using R statistical software and the GEO2R web tool. The Database for Annotation, Visualization and Integrated Discovery was used to identify the associated pathways, and for functional annotation of the differentially expressed genes (DEGs). Protein-protein interactions of these DEGs were analyzed based on the Search Tool for the Retrieval of Interacting Genes database, and hub genes were visualized using Cytoscape software. An online Kaplan-Meier plotter survival analysis tool was utilized to evaluate the prognostic value of hub gene expression in patients with EC. Further analysis of an additional dataset from GEO (GSE21293) revealed that these genes were associated with infiltration and metastasis in EC. In addition, the Gene Expression Profiling Interactive Analysis tool was used to evaluate expression levels of hub genes in patients with EC for different pathological stages. The Ualcan analysis tool was used to evaluate the expression levels of hub genes for different histological types. Overall, ubiquitin conjugating enzyme E2 C, cyclin dependent kinase inhibitor 3, CDC28 protein kinase regulatory subunit 2, kinesin family member 20A (KIF20A) and RAD51 associated protein 1 (RAD51AP1) were upregulated in EC tissues compared with normal tissues, and upregulation of these genes was a poor prognostic factor for patients with EC, indicating that these genes may mediate EC cell infiltration and metastasis. Among the hub genes, KIF-20A had potential value for predicting the pathological stage of EC. KIF20A and RAD51AP1 were more informative biomarkers of esophageal squamous cell carcinoma. Further studies are required to explore the value of these genes in the treatment of EC.

## Introduction

Gastrointestinal cancer is a major health problem with 4.8 million new cases and 3.4 million mortality cases reported worldwide in 2018 (1). Esophageal cancer (EC) is an aggressive malignancy originating in the gastrointestinal tract, with the eighth highest cancer incidence worldwide, and is the sixth most frequent cause of cancer-associated mortality between 1995 and 2009 (2). EC is more common in Asia than in other parts of the world (1), and EC incidence rate in China ranks first in the world, and is >35.0% higher than in the rest of the world. EC is categorized into esophageal squamous cell carcinoma (ESCC) and esophageal adenocarcinoma (EAC). ESCC is the major type of EC found in China (3). Currently, the primary treatment for EC is surgery; however, due to the absence of early symptoms, most patients are diagnosed with advanced EC, which results in high mortality rate (3). The main risk factors for EC are cigarette smoking, tobacco chewing, alcohol consumption, high body mass index and low fruit consumption (4). In addition, EC involves changes in multiple gene expression patterns (5). Therefore, understanding the mechanisms of initiation of EC and potential prognostic molecular markers are urgently required to improve the outcomes for patients with EC.

The present study aimed to identify differentially expressed genes (DEGs) between normal esophageal and EC tissues, and to subsequently classify these DEGs based on motifs and modules at a functional level according to Gene Ontology (GO) and Kyoto Encyclopedia of Genes and Genomes (KEGG) pathway analysis, which could provide a model suggesting mechanisms of how these DEGs contribute to EC. A protein-protein interaction (PPI) network was used to identify hub genes in EC. The present study identified which hub genes influenced the prognosis of EC, and explored the association among hub genes with prognostic value and invasive, metastatic, pathological and histological characteristics of EC. Finally, biomarkers that may be involved in the occurrence and development of EC were identified, and provide a basis for further research.

## Materials and methods

### 

#### Data source

To explore DEGs between EC and normal tissues, gene expression data were retrieved from the esophagus dataset (20160128) from The Cancer Genome Atlas (TCGA) database (http://tcga-data.nci.nih.gov; accessed, November 10, 2018). The ‘RTCGAToolbox’ package (version 1.0) in the R software environment was used to download quantitative gene expression data and clinical characteristics of patients with EC in the TCGA database (6). The running time was ‘20160128’ (updated version of esophageal cancer data in TCGA database when analyzing data). A total of 1,413 datasets of human ECs were retrieved from the Gene Expression Omnibus (GEO) database (https://www.ncbi.nlm.nih.gov/geo/). Following reading and screening, the three gene expression datasets GSE23400 (7), GSE20347 (8) and GSE5364 (9) were selected for further analysis. Among these, GSE23400 and GSE5364 were based on the GPL96 platform [(HG-U133A) Affymetrix Human Genome U133A Array; Affymetrix; Thermo Fisher Scientific, Inc.], and GSE20347 was based on the Agilent GPL571 platform [(HG-U133A_2) Affymetrix Human Genome U133A 2.0 Array; Affymetrix; Thermo Fisher Scientific, Inc.].

#### Processing of DEG expression data

The ‘RTCGAToolbox’ was used to evaluate DEGs between EC and normal tissue samples using data from the TCGA database (6), and genes that met the cutoff criteria [adjusted P-value =0.05; |log fold change (FC)| ≥2.0] were considered to be significant DEGs.

The GEO2R online analysis tool (https://www.ncbi.nlm.nih.gov/geo/geo2r/) was used to evaluate DEGs between EC and normal tissue samples in data from the GEO database. Due to the small sample size, the parameters used to indicate significant DEGs were adjusted P-value =0.05 and |log FC| ≥1.0.

Finally, a Venn diagram web tool (http://bioinformatics.psb.ugent.be/webtools/Venn/) was used to identify the intersection of DEGs between the TCGA and GEO datasets. The present study first identified the intersection of the three GEO datasets, and then cross-identified the DEGs common to the TCGA and GEO datasets. These intersecting genes were considered to be the DEGs between EC and normal tissue samples.

#### GO and KEGG pathway analysis of DEGs

GO (10) and KEGG (11) pathway enrichment analysis of DEGs was performed using the Database for Annotation, Visualization and Integrated Discovery (DAVID; http://david.ncifcrf.gov/) (12). P<0.05 was considered to indicate a statistically significant difference.

#### PPI network construction and hub gene identification

PPI information was analyzed using the STRING database (http://string-db.org/) (13). PPI pairs were extracted based on the condition of medium confidence >0.4. The PPI network was then visualized using Cytoscape software (www.cytoscape.org/; version 22.03.6.1) (14). In the present study, genes with a connectivity >40 were considered to be hub genes. Plug-in molecular complex detection (MCODE) was used to screen PPI networks (15). The maximum depth (value =100), the degree cutoff (value =10), the node score (value =0.2) and the k score (value =2) were set as the cutoff criteria.

#### Survival analysis of expression of hub genes

To explore the association between hub gene expression and prognosis of EC, the present study used a Kaplan-Meier plotter tool (http://kmplot.com/analysis/) to assess the impact of differential expression of the hub genes on the prognosis of EC (16). The Kaplan-Meier plotter database data are mainly derived from GEO (Affymetrix microarray only), European Genome-phenome Archive (EGA) and TCGA. Overall survival (OS) and relapse-free survival (RFS) were selected as the main indicators of prognostic assessment. The mean expression of all probes of the same gene was calculated as the expression of each gene. All possible cutoff values between the lower and upper quartiles were computed and the best performing threshold was used as the cutoff. The log-rank test was used to analyze OS and RFS. A log-rank P<0.05 was considered to indicate a statistically significant difference.

#### Invasion analysis of prognostic hub genes

To explore the role of the hub DEGs in EC invasion, an additional dataset, GSE21293 (17), was evaluated from the GEO database (https://www.ncbi.nlm.nih.gov/geo/). This dataset contained gene expression data for invasive and non-invasive EC cells. Fragments per kilobase million values for ubiquitin conjugating enzyme E2 C (UBE2C), cyclin dependent kinase inhibitor 3 (CDKN3), CDC28 protein kinase regulatory subunit 2 (CKS2), kinesin family member 20A (KIF20A) and RAD51 associated protein 1 (RAD51AP1) genes were log_2_ transformed and the resulting heatmap was constructed using HemI software (version 1.0) (18). Graph design and data anlysis were performed using GraphPad Prism 5.0 (GraphPad Software, Inc.). Student's t test was used to analyze difference between two groups. P<0.05 was considered to indicate a statistically significant difference.

#### Pathological staging analysis of prognostic hub gene expression

To explore the expression levels of hub genes across different stages of EC, the Gene Expression Profiling Interactive Analysis tool (http://gepia.cancer-pku.cn/) was used to analyze the hub gene expression data from the TCGA EC dataset, which contained clinical staging information (19).

#### Histological subtype analysis of prognostic hub gene expression

To analyze the differential expression of the prognostic hub genes in different histological subtypes of EC, the interactive website Ualcan (http://ualcan.path.uab.edu/analysis.html) was used to analyze the hub gene expression data from the TCGA EC dataset, which contained histological subtype information (20).

## Results

### 

#### Identification of DEGs

**Figure 1.**
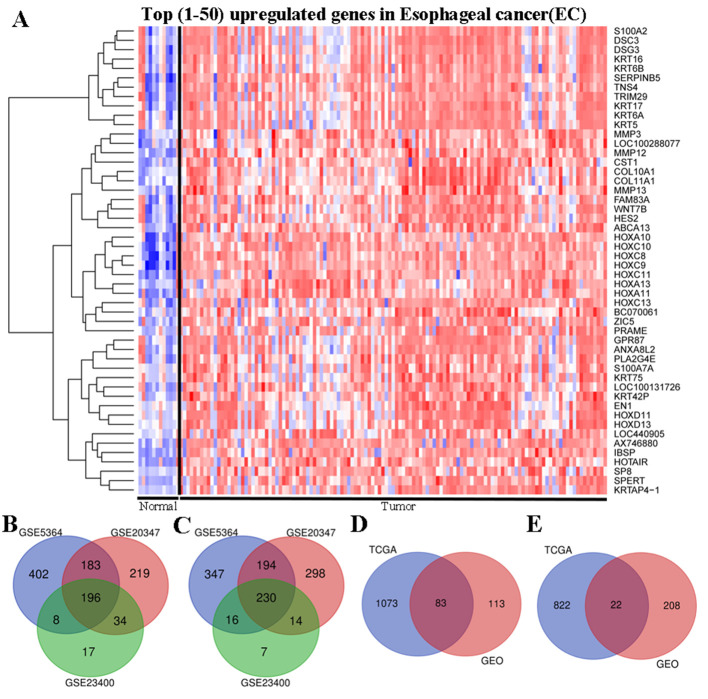
Identification of DEGs. (A) Heat map of the top 50 DEGs between EC and normal tissue samples from the TCGA dataset. Red indicates upregulation, and blue represents downregulation. Venn diagram of DEGs indicating commonly (B) upregulated genes and (C) downregulated genes among all three GEO datasets. Venn diagram of DEGs indicating commonly (D) upregulated genes and (E) downregulated genes among the GEO and TCGA datasets. DEG, differentially expressed gene; EC, esophageal cancer; GEO, Gene Expression Omnibus; TCGA, The Cancer Genome Atlas.

To identify DEGs between EC and normal tissues, the present study used two public gene expression databases. Initially, the TCGA database EC data (including 89 EAC samples and 11 normal esophageal samples) were used to identify DEGs. It was observed that samples from the same tissue type aggregated into a single group, and each tissue type exhibited one or more unique tissue-specific mRNA blocks. The top 50 genes were significantly upregulated in EC tissues compared with in normal tissues (Fig. 1A).

Subsequently, gene expression in three microarray datasets [GSE5364 (7), GSE20347 (8) and GSE23400 (9)] from the GEO database was explored. Among these, GSE5364 contained 16 EC and 13 normal samples, GSE20347 contained 17 EC and 17 normal samples, and GSE23400 contained 53 EC and 53 normal samples (Table I). Due to the small sample size, the criteria P<0.05 and |log FC| ≥1 were used to identify DEGs. A total of 1,576 DEGs were identified from the GSE5364 dataset, including 789 upregulated and 787 downregulated genes. In the GSE20347 dataset, 1,368 DEGs were identified; 632 genes were upregulated and 736 genes were downregulated. In the GSE23400 dataset, 522 DEGs were identified, including 255 upregulated genes and 267 downregulated genes. To avoid bias from individual studies, a Venn diagram was constructed to identify genes that were commonly upregulated and downregulated among the three datasets. The present study identified 426 DEGs that were significantly differentially expressed among all three datasets, of which 196 were significantly upregulated (Fig. 1B) and 230 were downregulated (Fig. 1C).

Based on the significance criteria of P<0.05 and |log FC| ≥2, a total of 2,000 DEGs were identified, including 1,156 upregulated genes and 844 downregulated genes in TCGA datasets (Fig. 1D-E). A comprehensive analysis of the data from TCGA and GEO was performed using Venn diagram analysis to identify the intersection of all DEGs. The present study identified 105 genes that were commonly significantly differentially expressed between EC and normal tissues in the two databases, of which 83 were significantly upregulated (Fig. 1D) and 22 were downregulated (Fig. 1E).

#### Functional enrichment analysis of DEGs

**Figure 2.**
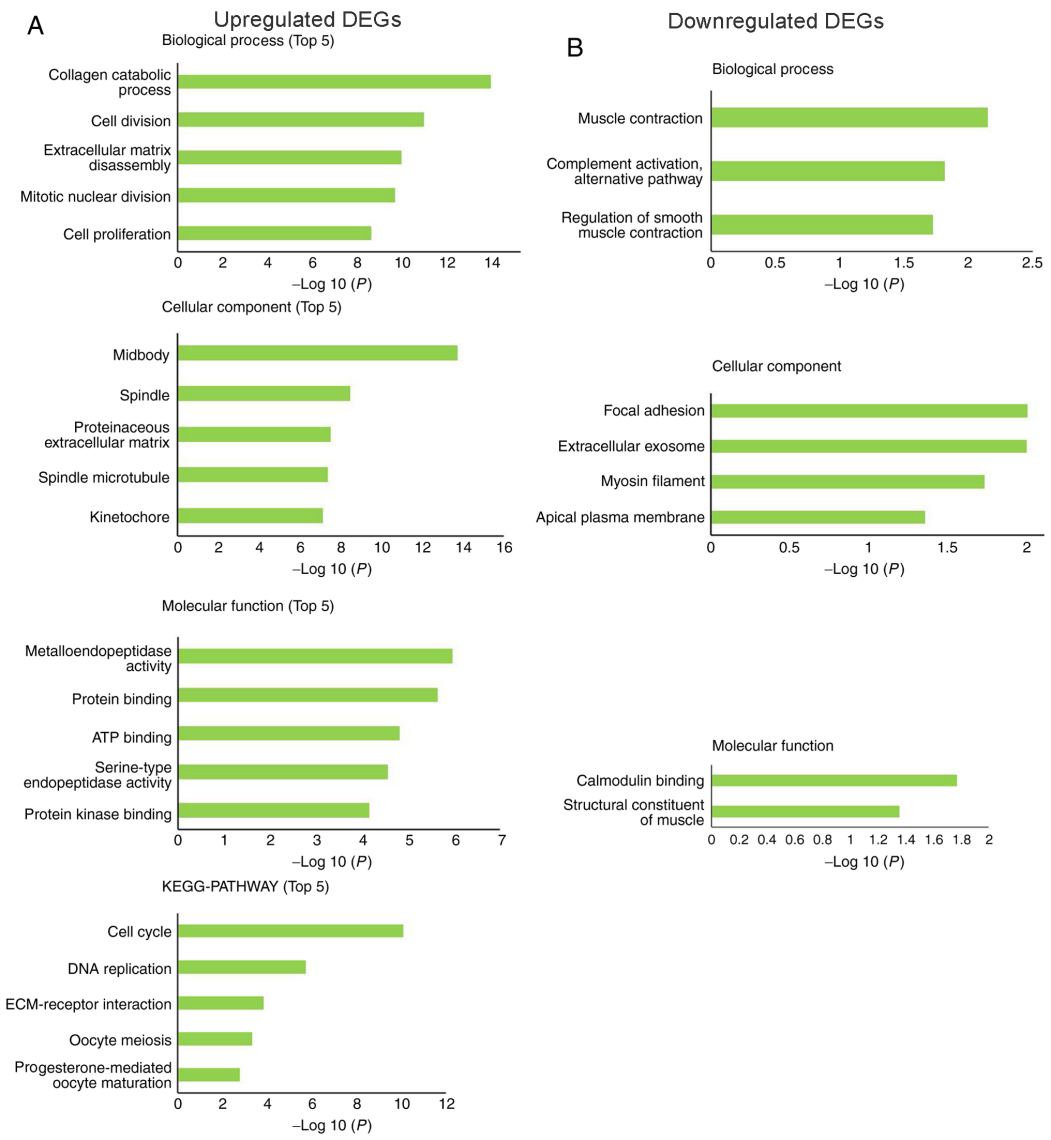
GO functional and KEGG pathway enrichment analyses of DEGs. The DAVID web tool was used to perform GO functional and KEGG pathway enrichment analyses of DEGs. (A) Top five most significant GO functional and KEGG pathway enrichment of the upregulated DEGs (P<0.001). (B) GO functional enrichment of downregulated DEGs (P<0.05). DEG, differentially expressed gene; ECM, extracellular matrix; GO, Gene Ontology; KEGG, Kyoto Encyclopedia of Genes and Genomes.

To investigate the effects of DEGs on the occurrence of EC at a functional level, the DAVID web tool was used to perform GO functional and KEGG pathway enrichment analysis of the DEGs. The enriched GO terms were divided into cellular components (CCs), biological processes (BPs) and molecular functions (MFs). The BPs associated with upregulated DEGs were ‘collagen catabolic process’, ‘cell division’, ‘extracellular matrix disassembly’, ‘mitotic nuclear division’ and ‘cell proliferation’. For CCs, the upregulated DEGs were mainly enriched in ‘midbody’, ‘spindle’, ‘proteinaceous extracellular matrix’, ‘spindle microtubule’ and ‘kinetochore’. MF analysis demonstrated that the upregulated DEGs were mainly enriched in ‘metalloendopeptidase activity’, ‘protein binding’, ‘ATP binding’, ‘serine-type endopeptidase activity’ and ‘protein kinase binding’. KEGG pathway analysis revealed that the upregulated DEGs were mainly enriched in ‘cell cycle’, ‘DNA replication’, ‘ECM-receptor interaction’, ‘oocyte meiosis’ and ‘progesterone-mediated oocyte maturation’ pathways (Fig. 2A).

The downregulated DEGs were mainly enriched in BPs, including ‘muscle contraction’, ‘complement activation, alternative pathway’ and ‘regulation of smooth muscle contraction’. The downregulated DEGs were significantly enriched in the CC terms ‘focal adhesion’, ‘extracellular exosome’, ‘myosin filament’ and ‘apical plasma membrane’. Enrichment analysis of MF terms revealed that downregulated DEGs were mainly enriched in ‘calmodulin binding’ and ‘structural constituent of muscle’. There was no significant KEGG pathway enrichment for the downregulated DEGs (Fig. 2B).

#### PPI network construction and hub gene identification

**Figure 3.**
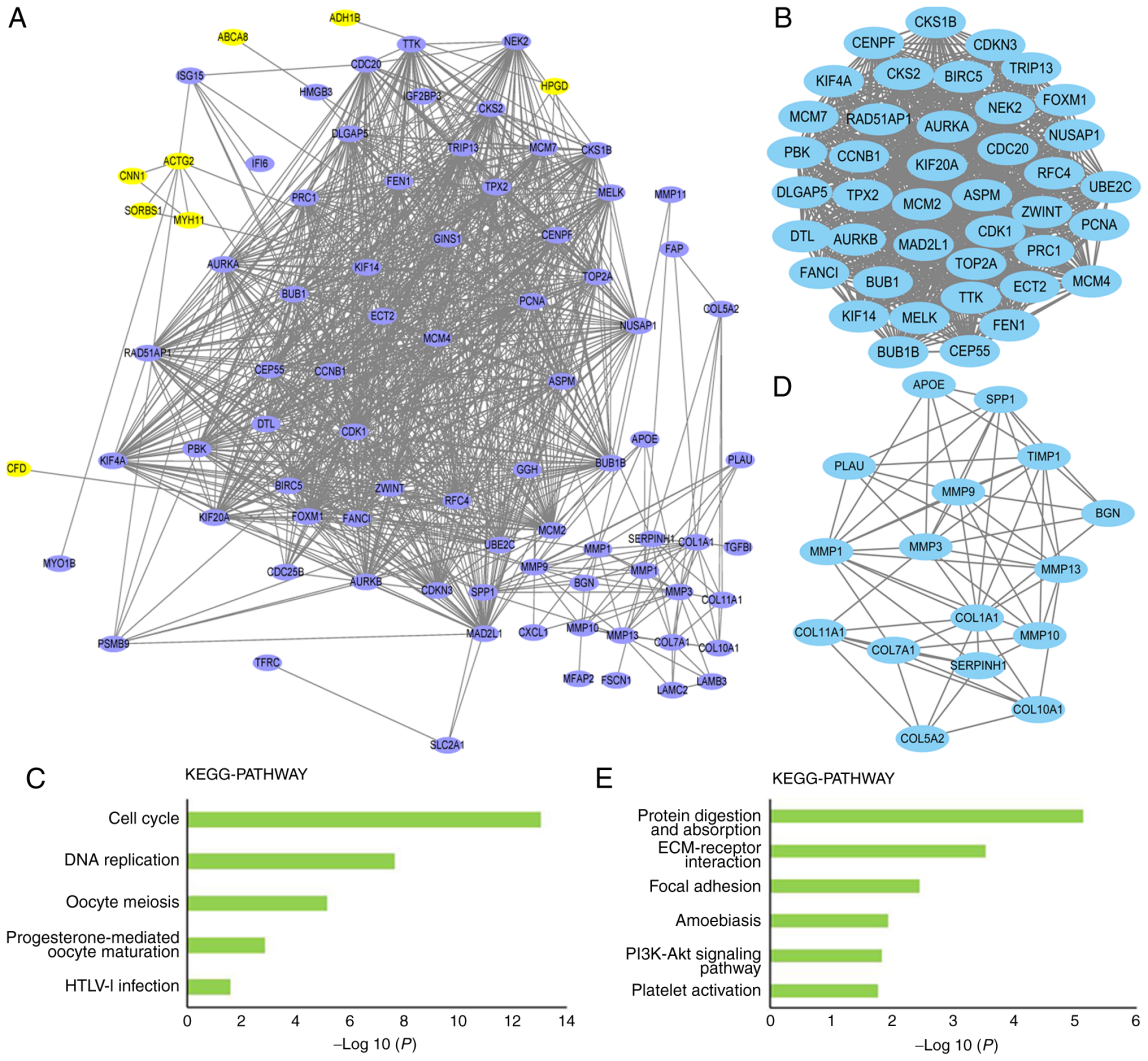
Protein-protein interaction network. (A) Protein-protein interaction network was constructed using DEGs. Purple nodes represent upregulated DEGs and yellow nodes represent downregulated DEGs. (B and C) Top two clusters of highly interconnected lysine-succinylated protein networks. Interaction network of protein-protein interaction network were analyzed using the MCODE plug-in toolkit in the Cytoscape software (version 3.0.1). (B) Module 1. MCODE score, 40.7; nodes, 41; edges, 814. (C) Significantly enriched KEGG pathways of module 1 DEGs. (D) Module 2. MCODE score, 8.4; nodes, 16; edges, 63. (E) Significantly enriched KEGG pathways of module 2 DEGs. DEG, differentially expressed gene; ECM, extracellular matrix; HTLV–I, human T-lymphotropic virus 1; KEGG, Kyoto Encyclopedia of Genes and Genomes; MCODE, molecular complex detection.

Cytoscape software was used to predict protein interactions among DEGs. The PPI network comprised 82 nodes and 982 edges (Fig. 3A). After analyzing the degree of connectivity in the PPI network, genes with a connectivity degree >40 were considered to be hub genes. There were 36 hub genes, and all of them were upregulated genes in EC (Table II).

Screening of the PPI network using MCODE identified two modules. In module 1, the MCODE score was 40.7, including 41 nodes and 814 edge connection lines (Fig. 3B). To investigate the effect of modules on the occurrence of EC at a more functional level, DEGs within the modules were classified into KEGG terms. The DEGs in module 1 were mainly enriched in KEGG pathways such as ‘cell cycle’, ‘DNA replication’, ‘oocyte meiosis’, ‘progesterone-mediated oocyte maturation’ and ‘HTLV–I infection’ (P<0.05; Fig. 3C). The MCODE score in module 2 was 8.4, including 16 nodes and 63 edge lines (Fig. 3D), and the KEGG pathway enrichment analysis of module 2 mostly identified terms such as ‘protein digestion and absorption’, ‘ECM-receptor interaction’, ‘focal adhesion’, ‘amoebiasis’, ‘PI3K-Akt signaling pathway’ and ‘platelet activation’ (P<0.05; Fig. 3E).

#### Analysis of hub genes as prognostic indicators of survival in EC

**Figure 4.**
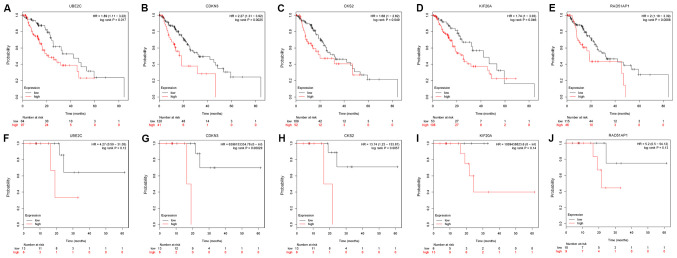
Kaplan-Meier overall survival analyses for hub genes expressed in EC. A total of five hub genes were identified to have an adverse effect on prognosis of overall survival for patients with EC. The Kaplan-Meier Plotter bioinformatics analysis platform was used to analyze the prognostic value of hub genes in the Gene Expression Omnibus and The Cancer Genome Atlas EC datasets. Overall survival of 161 patients with EC was determined according to (A) UBE2C, (B) CDKN3, (C) CKS2, (D) KIF20A and (E) RAD51AP1 status using the online Kaplan-Meier Plotter tool. Relapse-free survival of 73 patients with esophageal cancer according the (F) UBE2C, (G) CDKN3, (H) CKS2, (I) KIF20A and (J) RAD51AP1 status in online Kaplan-Meier Plotter (HR>1). CDKN3, cyclin dependent kinase inhibitor 3; CKS2, CDC28 protein kinase regulatory subunit 2; EC, esophageal cancer; HR, hazard ratio; KIF20A, kinesin family member 20A; RAD51AP1, RAD51 associated protein 1; UBE2C, ubiquitin conjugating enzyme E2 C.

To determine whether these DEGs were associated with survival of patients with EC, the DEGs were submitted to the Kaplan-Meier plotter bioinformatics analysis platform. Kaplan-Meier plotter database data is mainly derived from GEO (Affymetrix microarray only), EGA and TCGA. All possible cutoff values between the lower and upper quartiles were computed, and the best performing threshold was used as a cutoff. The log-rank test was used to analyze OS (161 EC samples) and RFS (73 EC samples). The OS prognosis of patients with EC with high expression levels of UBE2C (Fig. 4A), CDKN3 (Fig. 4B), CKS2 (Fig. 4C), KIF20A (Fig. 4D) and RAD51AP1 (Fig. 4E) was worse than that of patients with low expression levels of these genes (P<0.05). There was no significant prognostic value identified for the other hub genes (all P>0.05). To further investigate the impact of these five hub genes on the prognosis of patients with EC, the association between the expression of these genes and RFS was analyzed. The results demonstrated that UBE2C expression (Fig. 4F) was not associated with RFS, and higher expression levels of CDKN3 (Fig. 4G) and CKS2 (Fig. 4H) were significantly associated with shorter RFS in patients with EC. However, the expression levels of KIF20A (Fig. 4I) and RAD51AP1 (Fig. 4J) were not associated with RFS in patients with EC.

#### Influence of the five prognostic hub genes on invasion of EC

**Figure 5.**
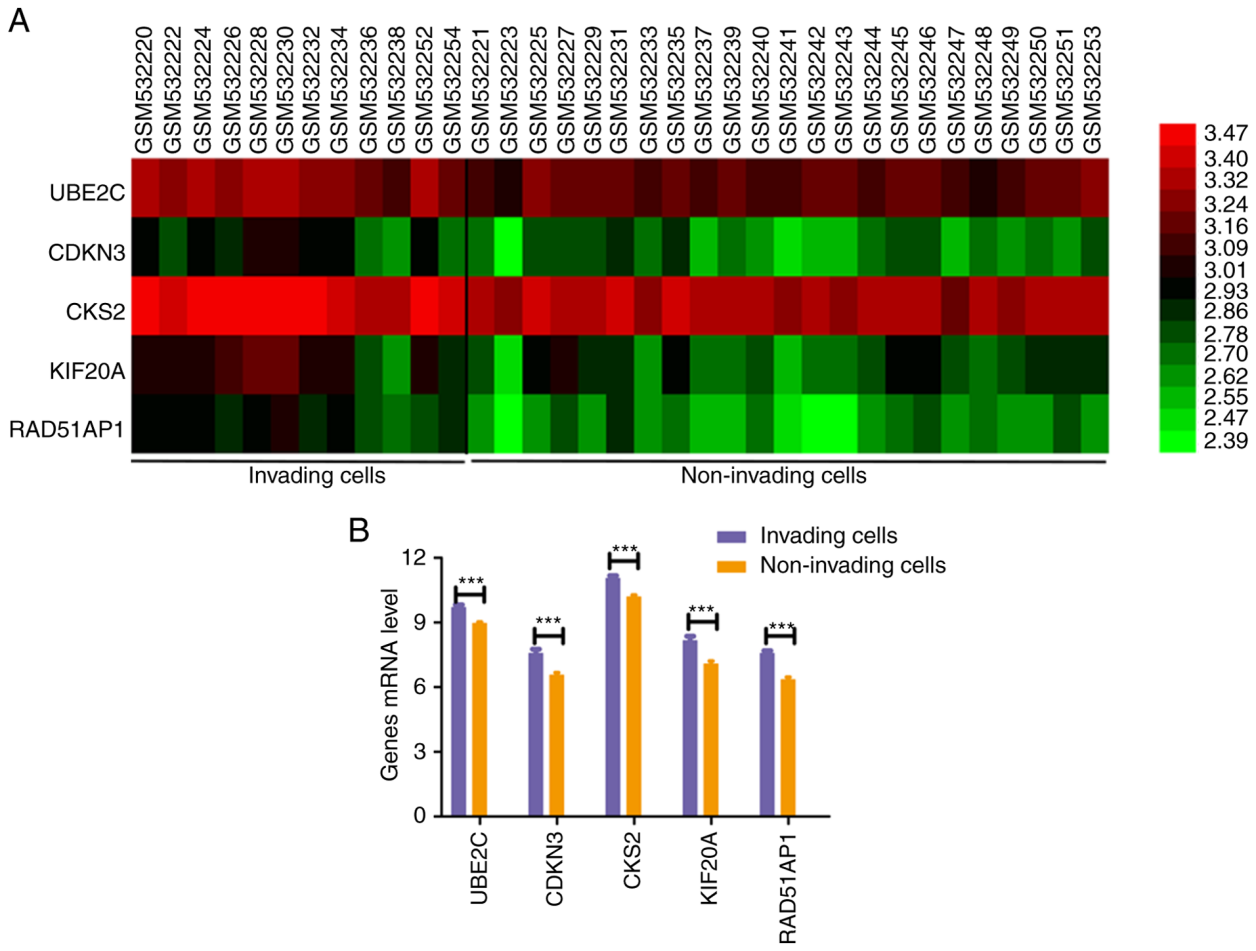
Role of five prognostic hub genes in the migration and invasion of esophageal cancer. (A) Gene expression of UBE2C, CDKN3, CKS2, KIF20A and RAD51AP1 in invading and non-invading cells based on the Gene Expression Omnibus expression dataset GSE21293. Fragments per kilobase million values of UBE2C, CDKN3, CKS2, KIF20A and RAD51AP1 genes were log_2_ transformed and the resulting heatmap was constructed using HemI software. (B) Bar chart indicating expression levels of UBE2C, CDKN3, CKS2, KIF20A and RAD51AP1 in the invading cells (n=12) compared with in the non-invading cells. CDKN3, cyclin dependent kinase inhibitor 3; CKS2, CDC28 protein kinase regulatory subunit 2; KIF20A, kinesin family member 20A; RAD51AP1, RAD51 associated protein 1; UBE2C, ubiquitin conjugating enzyme E2 C. ***P<0.001.

Poor prognosis of EC is closely associated with early invasion and metastasis (21). To determine if the five prognostic hub genes mediated EC migration and invasion, differences in expression levels of these genes in invading and non-invading EC cells from a GEO microarray dataset (GSE21293) were analyzed (17). Primary esophageal cells were established from ESCC surgical specimens (n=35). The present study revealed that UBE2C, CDKN3, CKS2, KIF20A and RAD51AP1 were significantly differentially expressed between invading and non-invading EC cells (Fig. 5A). UBE2C, CDKN3, CKS2, KIF20A and RAD51AP1 were upregulated in the invading cells (n=12) compared with the non-invading cells using Student's t test (Fig. 5B). These results indicated that these hub genes may mediate the migration and invasion of EC.

#### Association of the five prognostic hub genes with pathological staging of EC

**Figure 6.**
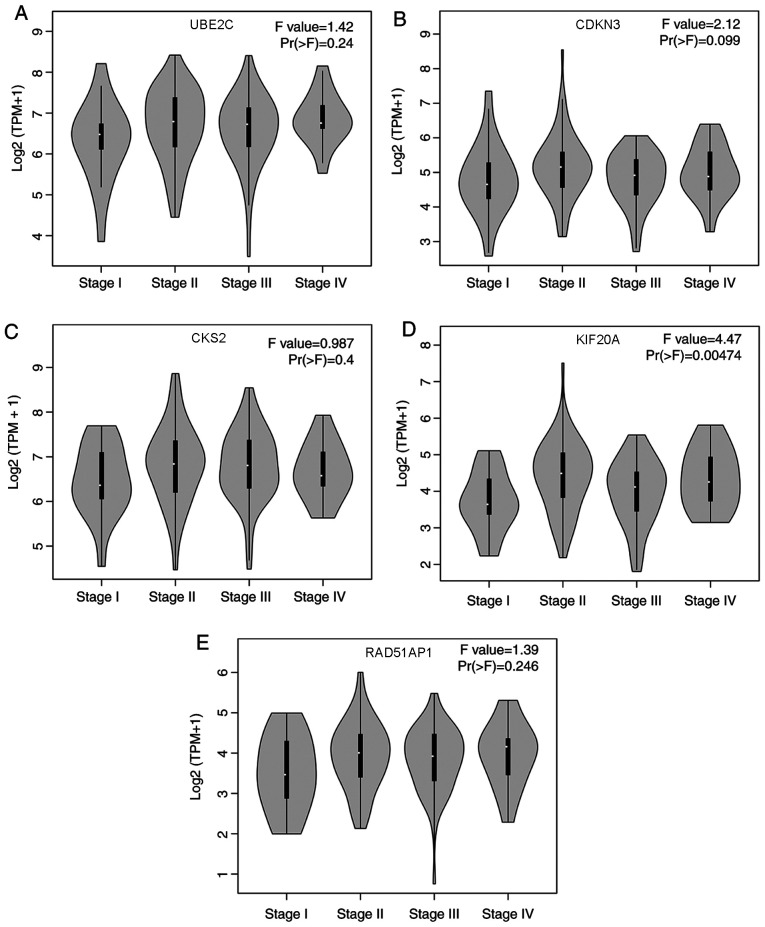
Association between clinical staging of EC and expression levels of the five hub genes. Differential expression of (A) ubiquitin conjugating enzyme E2 C, (B) cyclin dependent kinase inhibitor 3, (C) CDC28 protein kinase regulatory subunit 2, (D) kinesin family member 20A and (E) RAD51 associated protein 1 in different pathological stages of EC based on the Gene Expression Profiling Interactive Analysis database (including The Cancer Genome Atlas EC samples; n=182). EC, esophageal cancer. The Pr (>F) value is equivalent to the P-value, and Pr (>F) <0.05 is considered statistically significant.

Pathological stage is a major indicator of the progression of EC (3). The present study ascertained the association between expression levels of the five prognostic hub genes and pathological staging of EC. There were no significant differences identified for expression of UBE2C (Fig. 6A), CDKN3 (Fig. 6B) or CKS2 (Fig. 6C) in different pathological stages of EC (P>0.05). However, KIF20A expression was associated with different pathological stages of EC, and upregulation of KIF20A was closely associated with advanced EC stage (P<0.05; Fig. 6D). RAD51AP1 expression exhibited a non-significant trend of difference in different pathological stages of EC (P>0.05; Fig. 6E). Therefore, among these five hub genes, KIF20A expression may be used to predict the pathological stage of EC.

#### Differential expression of five prognostic hub genes in different histological subtypes of EC

**Figure 7.**
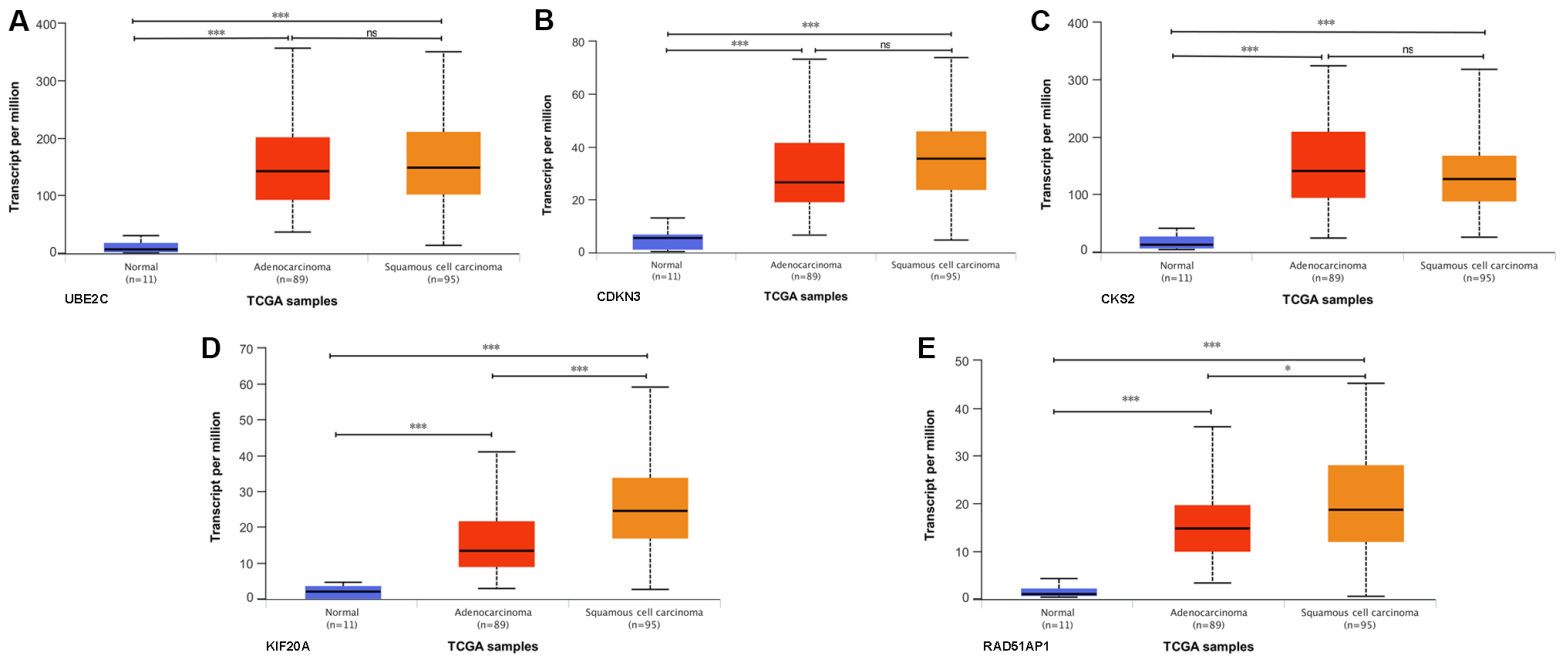
Expression levels of the five prognostic hub genes in different histological subtypes of esophageal cancer. Expression levels of (A) ubiquitin conjugating enzyme E2 C, (B) cyclin dependent kinase inhibitor 3, (C) CDC28 protein kinase regulatory subunit 2, (D) kinesin family member 20A and (E) RAD51 associated protein 1 in different histological subtypes based on the Ualcan database (including TCGA esophagus samples; n=195). *P<0.05 and ***P<0.001, as indicated. ns, not significant; TCGA, The Cancer Genome Atlas.

To determine if the expression of the five prognostic hub genes differed in different histological subtypes of EC, the expression levels of these genes in ESCC and EAC were analyzed based on the TCGA database. The expression levels of UBE2C (Fig. 7A), CDKN3 (Fig. 7B), CKS2 (Fig. 7C), KIF20A (Fig. 7D) and RAD51AP1 (Fig. 7E) in EAC and ESCC were significantly higher than those in normal tissues. The expression levels of UBE2C, CDKN3 and CKS2 exhibited no significant difference between different histological EC subtypes. Higher expression levels of KIF20A and RAD51AP1 were identified in ESCC compared with EAC. KIF20A and RAD51AP1 may represent more specific molecular targets for ESCC than EAC.

## Discussion

In the present study, through comprehensive analysis of EC gene expression profiles in the TCGA database and three datasets from the GEO database, a total of 105 DEGs were identified between EC and normal tissues. GO and KEGG pathway enrichment analyses were conducted to identify functional processes and pathways that may be mediated by these genes. GO enrichment analysis revealed that upregulated DEGs were mainly involved in ‘collagen catabolic process’, located in the ‘midbody’ and promoted ‘metalloendopeptidase activity’. KEGG pathway analysis revealed that the upregulated DEGs were mainly enriched in the ‘cell cycle’, and downregulated DEGs were mainly involved in ‘muscle contraction’, located in ‘focal adhesion’ and promoted ‘calmodulin binding’. These DEGs were also associated with the proliferation of tumor cells and cell matrix remodeling, which revealed that cell cycle disorders, and invasion and migration were important mechanisms leading to the development of EC. A total of 36 genes were identified as hub genes in PPI network analysis. Two significant modules of the network were identified and analyzed at a functional level to identify KEGG pathways associated with the occurrence of EC. The KEGG pathways identified included the ‘PI3K-Akt pathway’, which involves a series of important processes in EC, including repressing cell proliferation and tumor growth *in vitro* and *in vivo*, and regulation of a stem cell-like population (22,23). Based on Kaplan-Meier plotter analysis, upregulation of UBE2C, CDKN3, CKS2, RAD51AP1 and KIF20A was significantly associated with shorter OS in patients with EC, and higher expression levels of CDKN3 and CKS2 were significantly associated with shorter RFS in patients with EC. However, the mechanisms by which these DEGs were associated with the occurrence of EC remains unclear. Therefore, the present study evaluated the association between the expression levels of these DEGs and other clinical parameters of EC. It was identified that the five prognostic hub genes may mediate EC migration and invasion. KIF20A may have potential value as a predictive biomarker for the pathological staging of EC. Compared with in EAC, KIF20A and RAD51AP1 were upregulated in ESCC.

UBE2C targets abnormal or short-lived protein degradation during protein modification, and is a key modulator in controlling cell proliferation (24,25). Upregulation of UBE2C is considered to be a potential molecular marker for the prognosis of breast cancer and advanced colon cancer with liver metastases (26,27). Additionally, UBE2C is upregulated in EC, where it can target and regulate cyclin B1, control the cell cycle of EC cells and affect EC cell proliferation (28). The present study demonstrated that UBE2C was upregulated in EC, and that upregulation of UBE2C was an adverse prognostic indicator for patients with EC. At the mechanistic level, a recent study found that UBE2C is involved in the antiproliferative and apoptosis-regulating effects of ECRG4 augurin precursor (ECRG4) in EC cells (29). ECRG4 inhibits the proliferation of EC cells by downregulating the expression levels of UBE2C via the NF-κB signaling pathway (29). Therefore, UBE2C may be a prognostic factor and potential therapeutic target for EC.

CDKN3 is a member of the unspecific protein phosphatase family and interacts with CDK2 kinase to regulate the cell cycle (30,31). CDKN3 expression can reflect the proliferative activity of cells (32). Upregulation of CDKN3 is considered to be a key factor in promoting tumor cell proliferation and malignant transformation in ovarian cancer, cervical cancer, lung adenocarcinoma and leukemia, and is a potential molecular target for antitumor therapy (33–36). The present study revealed that CDKN3 was upregulated in EC tissues compared with in normal tissues, and patients with EC with high expression levels of CDKN3 tended to have a worse prognosis. To the best of our knowledge, the role of CDKN3 in EC remains unclear. CDKN3 may serve a role in the development of EC, and further studies should be performed to explore the value of CDKN3 as a therapeutic target in the treatment of EC.

CKS2 is a downstream target gene of p53, and p53 regulates the cell cycle by controlling CKS2 (37,38). Additionally, microRNA-7 and microRNA-26a can control the proliferation of tumor cells by targeting CKS2 (39,40), and Y-box binding protein 1 can regulate the cell cycle partly due to its role in downregulating CKS2 (41). CKS2 protein is highly expressed in EC, and is associated with higher histological grade, regional lymph node invasion, lymphatic vessel infiltration, advanced clinical stage and distant metastasis (42,43). High CKS2 expression is an unfavorable prognostic indicator for patients with EC (42,43). In the present study, the role of CKS2 in EC has been further elucidated.

KIF20A has been studied extensively in pancreatic carcinoma (44). Inhibition of the pancreatic carcinoma RAB6KIFL/KIF20A cell line by small interfering RNA targeting KIF20A can significantly inhibit the proliferation of this cell line (44). KIF20A serves a key role in malignant biological behaviors, including invasion and metastasis of pancreatic carcinoma cells (45). Upregulation of KIF20A has been demonstrated in numerous types of cancer, and is an independent prognostic factor for poor clinical outcomes for early-stage cervical squamous cell carcinoma, glioma and breast cancer (46–48). A KIF20A-targeted polypeptide vaccine, which induces a specific immune response of cytotoxic T lymphocytes, has been developed and has achieved good results in the treatment of advanced pancreatic carcinoma (49,50). Recently, it has been suggested that targeting KIF20A by immunotherapy may have potential therapeutic efficacy in glioma (51). In the present study, KIF20A was identified to be significantly upregulated in EC tissues, and KIF20A upregulation was associated with poor prognosis of EC and different clinical stages of EC, suggesting that KIF20A may be a key factor in the occurrence and development of EC, particularly ESCC.

RAD51AP1 is an auxiliary protein of RAD51 recombinase (RAD51). RAD51AP1 facilitates the repair of damaged DNA strands by binding to RAD51 (52,53). Inhibiting RAD51AP1 has been demonstrated to reduce the proliferation of cholangitis carcinoma cells. RAD51AP1 may serve a role in DNA repair and tumor cell proliferation (54). In malignant melanoma, high expression levels of RAD51AP1 may be an important molecular event involved in tumor invasion and metastasis (55). Additionally, DNA methylation in the RAD51AP1 promoter region may be associated with prostate carcinoma (56). The potential role of RAD51AP1 in the prognosis of EC merits further study.

In conclusion, a comprehensive bioinformatic analysis of DEGs that may be associated with the pathogenesis of EC was presented. A total of 105 DEGs and 36 hub genes were identified, and the hub genes UBE2C, CDKN3, CKS2, KIF20A and RAD51AP1 were associated with poor prognosis of patients with EC, and may be involved in invasion of EC. Differential expression of KIF20A was associated with different pathological stages of EC. The expression levels of KIF20A and RAD51AP1 in ESCC were higher than those in EAC, suggesting an EC subtype-specific expression pattern. The role of these genes in the progression of EC merits further investigation.

## Figures and Tables

**Table I.** Characteristics of the three microarray datasets retrieved from the Gene Expression Omnibus database.

	EC				
			
Dataset ID	EAC, n	ESCC, n	Total EC, n	Normal, n	Total number, n
GSE23400	0	53	53	53	106
GSE20347	0	17	17	17	34
GSE5364	Unclear	Unclear	16	13	19

EC, esophageal cancer; EAC, esophageal adenocarcinoma; ESCC, esophageal squamous cell carcinoma. Unclear, not clear in the dataset.

**Table II.** Hub genes with higher degree of connectivity.

Gene symbol	Degree
CDK1	46
PCNA	45
BIRC5	44
TOP2A	44
RFC4	44
CCNB1	44
CDC20	43
MAD2L1	43
FOXM1	43
AURKA	43
AURKB	42
UBE2C	42
TTK	42
NEK2	42
FEN1	42
BUB1B	42
CDKN3	42
MELK	42
TPX2	41
BUB1	41
CKS2	41
MCM2	41
ZWINT	41
PRC1	41
CENPF	41
DTL	41
PBK	41
ASPM	41
CEP55	41
MCM4	41
TRIP13	41
DLGAP5	40
CKS1B	40
KIF20A	40
NUSAP1	40
RAD51AP1	40

## Data Availability

All data generated or analyzed during this study are included in the published article.
